# Compensatory Vasodilator Mechanisms in the Ophthalmic Artery of Endothelial Nitric Oxide Synthase Gene Knockout Mice

**DOI:** 10.1038/s41598-017-07768-7

**Published:** 2017-08-02

**Authors:** Caroline Manicam, Natalja Ginter, Huige Li, Ning Xia, Evgeny Goloborodko, Jenia Kouchek Zadeh, Aytan Musayeva, Norbert Pfeiffer, Adrian Gericke

**Affiliations:** 1 Department of Ophthalmology, University Medical Centre of the Johannes Gutenberg University Mainz, Mainz, Germany; 2 Institute of Pharmacology, University Medical Centre of the Johannes Gutenberg University Mainz, Mainz, Germany

## Abstract

Nitric oxide (NO) generated by endothelial nitric oxide synthase (eNOS) plays an important role in the maintenance of ocular vascular homeostasis. Therefore, perturbations in vascular NO synthesis have been implicated in the pathogenesis of several ocular diseases. We recently reported that eNOS contributes significantly to vasodilation of the mouse ophthalmic artery. Interestingly, dilatory responses were also retained in eNOS gene-deficient mice (eNOS−/−), indicating inherent endothelial adaptive mechanism(s) that act as back-up systems in chronic absence of eNOS to preserve vasorelaxation. Thus, this study endeavoured to identify the compensatory mechanism(s) in the ophthalmic artery of eNOS−/− mice employing isolated arterial segments and pharmacological inhibitors *in vitro*. Endothelium removal virtually abolished acetylcholine (ACh)-induced vasodilation, suggesting an obligatory involvement of the endothelium in cholinergic control of vascular tone. However, non-NOS and non-cyclooxygenase components compensate for eNOS deficiency *via* endothelium-derived hyperpolarizing factors (EDHFs). Notably, arachidonic acid-derived metabolites of the 12-lipoxygenase pathway were key mediators in activating the inwardly rectifying potassium channels to compensate for chronic lack of eNOS. Conclusively, endothelium-dependent cholinergic responses of the ophthalmic artery in the eNOS−/− mice are largely preserved and, this vascular bed has the ability to compensate for the loss of normal vasodilator responses solely *via* EDHFs.

## Introduction

Since the seminal discovery of nitric oxide (NO) as an endogenous signalling molecule in the cardiovascular system, its role in the maintenance of vascular homeostasis and vasorelaxation remains indispensable^1^. It is well-recognized that three nitric oxide synthase (NOS) isoforms comprising the neuronal NOS (nNOS), inducible NOS (iNOS) and endothelial NOS (eNOS), are responsible for the synthesis of NO from the amino acid L-arginine^2, 3^. The distribution of these three NOS isoforms varies depending on tissue types and species, with pleiotropic physiological roles, besides mediating vasodilation^4, 5^. Notably, derangement in NO synthesis in blood vessels predisposes to the development of various pathologies^6^. Likewise, the relevance of the NOS/ NO pathway in the regulation of normal vascular function in the ocular blood vessels has been described in the retina, ciliary arteries, ophthalmic artery and choroidal blood vessels in different species, including humans^7–11^. Correspondingly, the lack of NO in the ocular vasculature and optic nerve may lead to the pathogenesis of several disorders, namely glaucomatous optic neuropathy and diabetic retinopathy^12–15^. Hence, it is not surprising that upregulation of eNOS is reported to confer neuroprotection *via* vasodilation and elevated optic nerve head blood flow^15–18^.

On the other hand, accumulating evidence from non-ocular blood vessels has demonstrated that endothelium-dependent vasodilatory responses can be preserved in arteries *via* eNOS-independent back-up mechanisms that can step into the breach when NO production is dysregulated or absent^19–22^. These mechanisms may involve the other two NOS isoforms, as well as the putative endothelium-derived hyperpolarizing factors (EDHFs). In retrospect, our investigation has shown that the eNOS isoform contributes to the vasodilatory responses of the ophthalmic artery of wild type mice (C57Bl/6J). Additionally, we also found that the endothelium-dependent vasodilation of the ophthalmic artery is retained in eNOS−/− mice^23^. This was followed by a recent study of ours that delved further to demonstrate that apart from eNOS, an array of EDHFs and potassium ion channels contributes to the maintenance of vascular reactivity to acetylcholine (ACh) in the ophthalmic artery of wild type mice^24^. Therefore, it remains to be determined whether in chronic absence of eNOS, similar compensation occurs to maintain near-normal function in the ophthalmic artery.

This study is a sequel of our previous investigation to identify potential compensatory mechanisms employing a mouse model with eNOS gene deficiency (eNOS−/−). Mouse models are powerful and established tools to study detailed patho-mechanisms of human diseases, including ocular pathologies, because it allows specific genetic manipulation^25^. Correspondingly, these experiments were carried out in vessels from mice with targeted disruption of the eNOS gene because selective eNOS inhibitors are not available at present^20, 22^.

The mouse ophthalmic artery shares some important morphological and functional similarities with that of human’s. It has been elegantly shown in many studies that the ophthalmic artery emanates from the internal carotid artery in both humans and mice^25–27^, and that NO is a crucial modulator of vascular tone in both species^8, 23, 24^. Since the ophthalmic artery is one of the major blood suppliers of the eye^28^, any dysfunction in this arterial bed is implicated in the pathophysiology of an array of disorders, including but not only limited in the eye, such as transient retinal ischemia caused by ophthalmic artery occlusion, severe ipsilateral internal carotid artery stenosis or occlusion and, secondary collateral ophthalmic artery flow is an indication of impaired cerebral hemodynamics in stroke patients^29–32^.

Therefore, considering the functional relevance of the ophthalmic artery, we hypothesized that although the endothelial mediation of arteriolar responses to ACh may be altered in eNOS−/− mice, other endothelium-dependent mediators and/or potassium ion channel(s) would compensate for the chronic absence of NO, providing for the maintenance of responses to ACh. A two-pronged approach employing both pharmacological inhibitors as well as mice with targeted deletion of the eNOS gene was used in this study.

## Results

### Endothelium-dependent cholinergic vasodilatory responses of the ophthalmic artery of eNOS−/− mice

**Figure 1:**
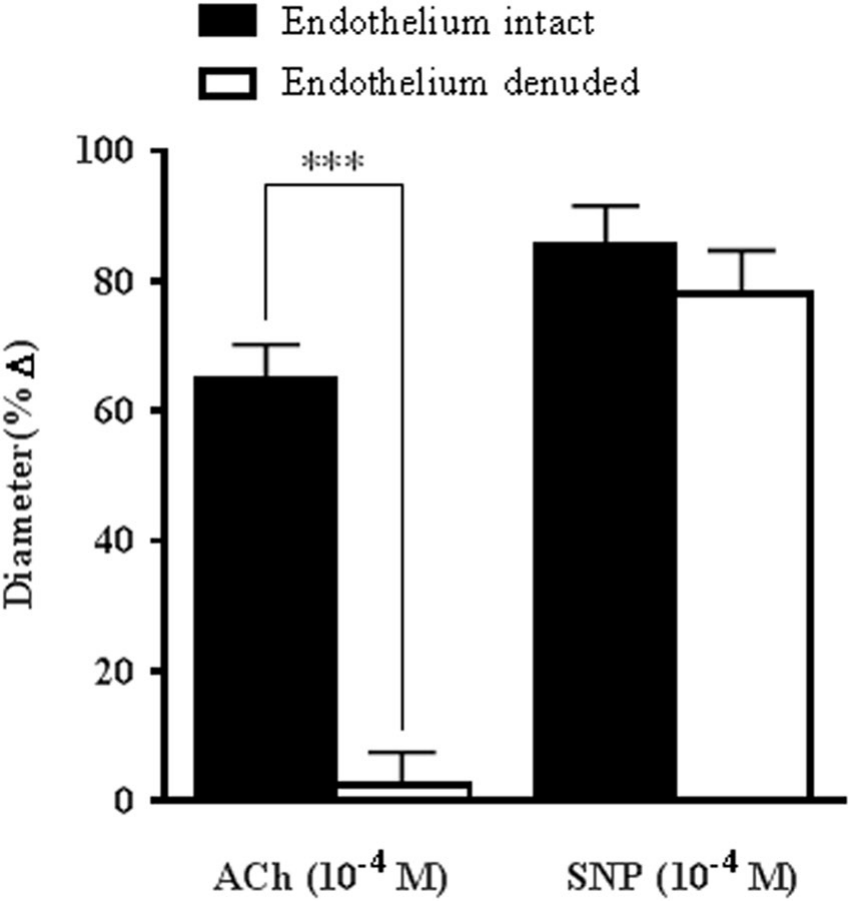
Role of endothelium in cholinergic vasodilatory responses of ophthalmic artery of eNOS−/− mice. Vasodilatory responses to ACh (10^−4^ M) were almost abolished in vessels denuded of endothelium. Conversely, vasodilation to exogenous NO donor, SNP (10^−4^ M) was preserved in both endothelium-denuded and -intact vessels. Values are expressed as mean ± standard error of the mean (s.e.m) (n = 6 per group; ***P < 0.001, endothelium-denuded versus endothelium-intact).

The role of endothelium in mediating vasodilatory responses was investigated in phenylephrine-preconstricted arteries with the endothelium-dependent agonist, ACh (10^−4^ M). Stimulation with ACh produced marked vasodilation in endothelium-intact vessels (64.87 ± 5.38%) but only negligible responses in endothelium-denuded vessels (2.37 ± 5.12%, P < 0.001). Conversely, dilator responses to an endothelium-independent stimulus, sodium nitroprusside (SNP; 10^−4^ M), were not impaired in the ophthalmic artery of eNOS−/− mice, as evidenced by almost similar responses in both endothelium-intact and -denuded vessels (85.41 ± 6.15% and 78.02 ± 6.66%, respectively), indicating that the reactivity of the smooth muscle cell layer is preserved albeit the removal of endothelium (Fig. 1).

### Role of NOS and cyclooxygenase (COX) in endothelium-dependent vasodilatory mechanisms

**Figure 2:**
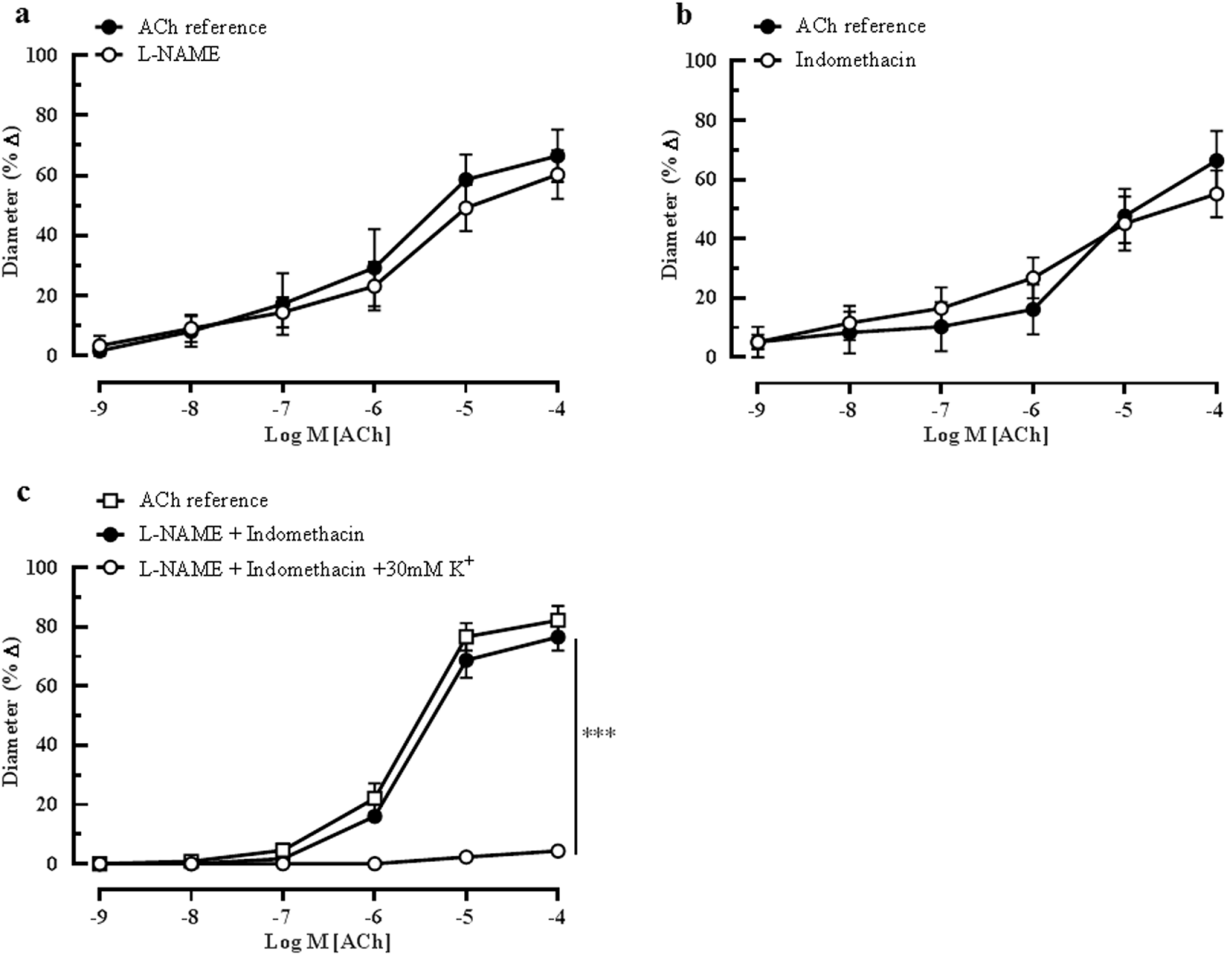
Effects of NOS and COX blockers on ACh-mediated vasodilatation. Endothelium-dependent responses of ophthalmic artery of eNOS−/− mice to ACh in the presence of (**a**) non-isoform-selective NOS inhibitor, L-NAME (10^−4^ M, n = 6) and (**b**) non-isoform-selective COX inhibitor, indomethacin (10^−5^ M, n = 6) exhibited no significant attenuation of dilation. (**c**) Similarly, combination blockade of both NOS and COX did not inhibit ACh-induced vasodilation. However, combined inhibition with L-NAME, indomethacin and a depolarizing concentration of potassium chloride solution (30 mM KCl) abolished non-NO, non-prostanoid-mediated relaxation to ACh (***P < 0.001, L-NAME and indomethacin *vs* L-NAME and indomethacin and KCl). Values are expressed as mean ± s.e.m.

To determine the functional role of other NOS isoforms in compensating for the lack of eNOS, concentration-response curves to ACh (10^−9^–10^−4^ M) were performed in the presence of the non-isoform-selective NOS inhibitor, L-NAME (10^−4^ M). Figure 2a shows that the inhibition of NOS had no significant effect on the ACh-mediated vasodilation of the ophthalmic artery (ACh reference: 66.50 ± 10.95% *vs* L-NAME: 60.29 ± 9.40% at 10^−4^ M). This result indicates that there is no NOS-related compensation in the absence of eNOS in the mouse ophthalmic artery. Similarly, the inhibition of COX with indomethacin (10^−5^M) did not exhibit significant attenuation of vasodilation (ACh reference: 66.39 ± 10.31% *vs* indomethacin: 55.14 ± 8.05%) (Fig. 2b), suggesting that COX metabolites do not play a role in the cholinergic vasodilatory responses to ACh in this vascular bed. The next protocol was carried out to determine the involvement of EDHF in mediating compensatory dilator responses during chronic absence of eNOS. Figure 2c presents data depicting the effect of potassium chloride (30 mM, KCl) on the dilator responses to cumulative administration of ACh (10^−9^–10^−4^M) in the presence of both inhibitors of NOS and COX. At this partially depolarizing concentration, KCl elicited significant abolishment of vasodilation (L-NAME+ indomethacin: 76.59 ± 14.63% *vs* L-NAME+ indomethacin +KCl: 4.33 ± 0.75%, P < 0.001), thereby confirming the predominant role of EDHF in ACh-mediated vasodilation in the ophthalmic artery of the eNOS−/− mice.

### Contribution of EDHF in mediating ACh-induced vasodilation

**Figure 3:**
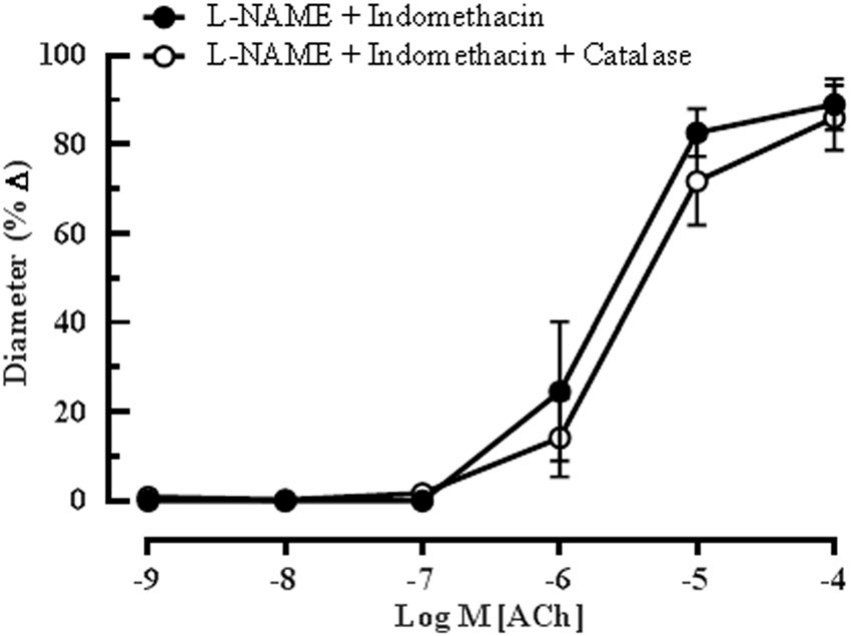
Catalase elicited negligible inhibitory effect on ACh-induced vasodilation. Blocking of H_2_O_2_, a putative EDHF, with catalase (1000 units/ml) in combination with L-NAME and indomethacin in the eNOS−/− mouse ophthalmic artery did not produce significant attenuation of vasodilation. Values are expressed as mean ± s.e.m (n = 6 per group).

To further investigate the possible mechanism(s) that compensates for the lack of eNOS, several pharmacological agents were employed to selectively inhibit the vasodilatory responses attributed to EDHF. All experiments were performed in the presence of both NOS and COX inhibitors. The inhibition of hydrogen peroxide (H_2_O_2_), a gasotransmitter implicated as putative EDHF^33^, with catalase (1000 units/ml) in combination of L-NAME and indomethacin elicited no inhibitory effect on the ACh-induced dilatation (L-NAME and indomethacin: 89.04 ± 17.27% *vs* Catalase: 85.99 ± 15.97%), as shown in Fig. 3.

**Figure 4:**
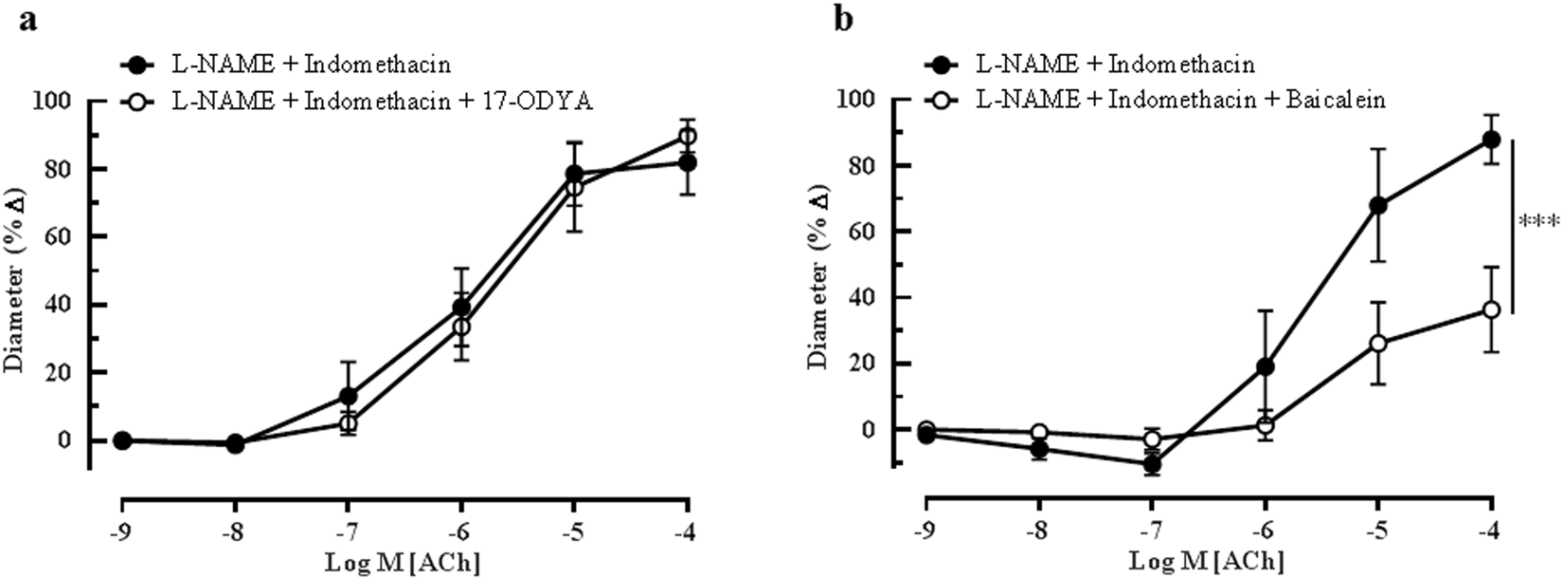
Endothelium-dependent relaxation of the ophthalmic artery attributed to EDHF *via* arachidonic acid metabolites. Concentration-response curves to ACh (10^−9^–10^−4^ M) in the presence of (**a**) CYP_450_ inhibitor, 17-ODYA and (**b**) LOX inhibitor, baicalein. Blocking of the CYP_450_ pathway conferred a negligible inhibitory effect on ACh-induced vasodilation, whereas, inhibition of the LOX pathway elicited significant attenuation of vasodilation. Values are expressed as mean ± s.e.m (n = 6 per group; ***P < 0.001, L-NAME and indomethacin *vs* L-NAME and indomethacin and baicalein).

**Figure 5:**
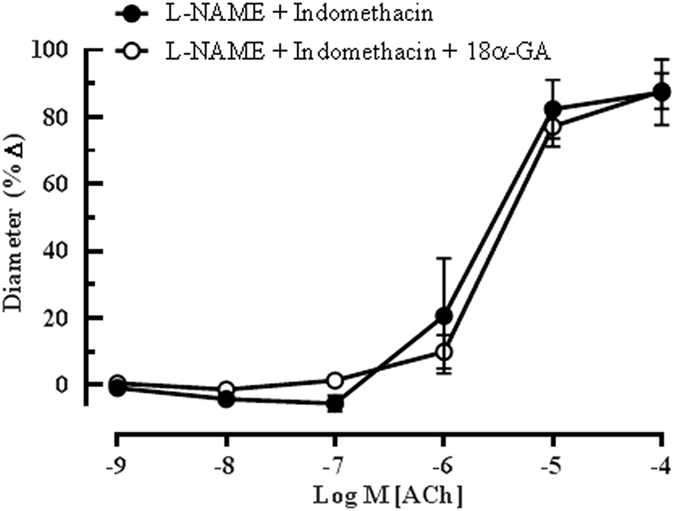
Effect of 18α-GA on EDHF-mediated relaxation of the ophthalmic artery from eNOS−/− mice to ACh. Incubation of the vessels with 18α-GA evoked no inhibitory effects on vasodilation, indicating null involvement of gap junctions in mediating EDHF-type responses in the ophthalmic artery. Values are expressed as mean ± s.e.m (n = 6 per group).

The role of arachidonic acid metabolites generates *via* the cytochrome P_450_ oxygenase (CYP450) and lipoxygenase (LOX) pathways as potential EDHFs in this vascular bed was investigated employing 17-ODYA and baicalein, respectively. Although 17-ODYA did not elicit any inhibitory effects on vasodilation of the ophthalmic artery (L-NAME and indomethacin: 81.95 ± 15.43% *vs* 17-ODYA: 89.76 ± 16.28%) (Fig. 4a), baicalein caused a significant reduction in relaxation to ACh (L-NAME and indomethacin: 87.95 ± 7.42% *vs* baicalein: 36.31 ± 12.94%, P < 0.001), demonstrating that the metabolites of 12/15-LOX are involved in the cholinergic compensatory mechanism (Fig. 4b). Previously, we have shown that gap junctions play an important functional role in the ophthalmic artery of wild type mice in concert with the arachidonic acid metabolites^24^. However, in the eNOS−/− mice, the inhibition of gap junctions with 18α -GA evoked only negligible inhibitory effect on vasodilation elicited by ACh (L-NAME and indomethacin: 87.43 ± 17.80% *vs* 18α -GA: 87.76 ± 16.96%), as depicted in Fig. 5.

### Involvement of potassium ion channels in endothelium-dependent vasodilation

**Figure 6:**
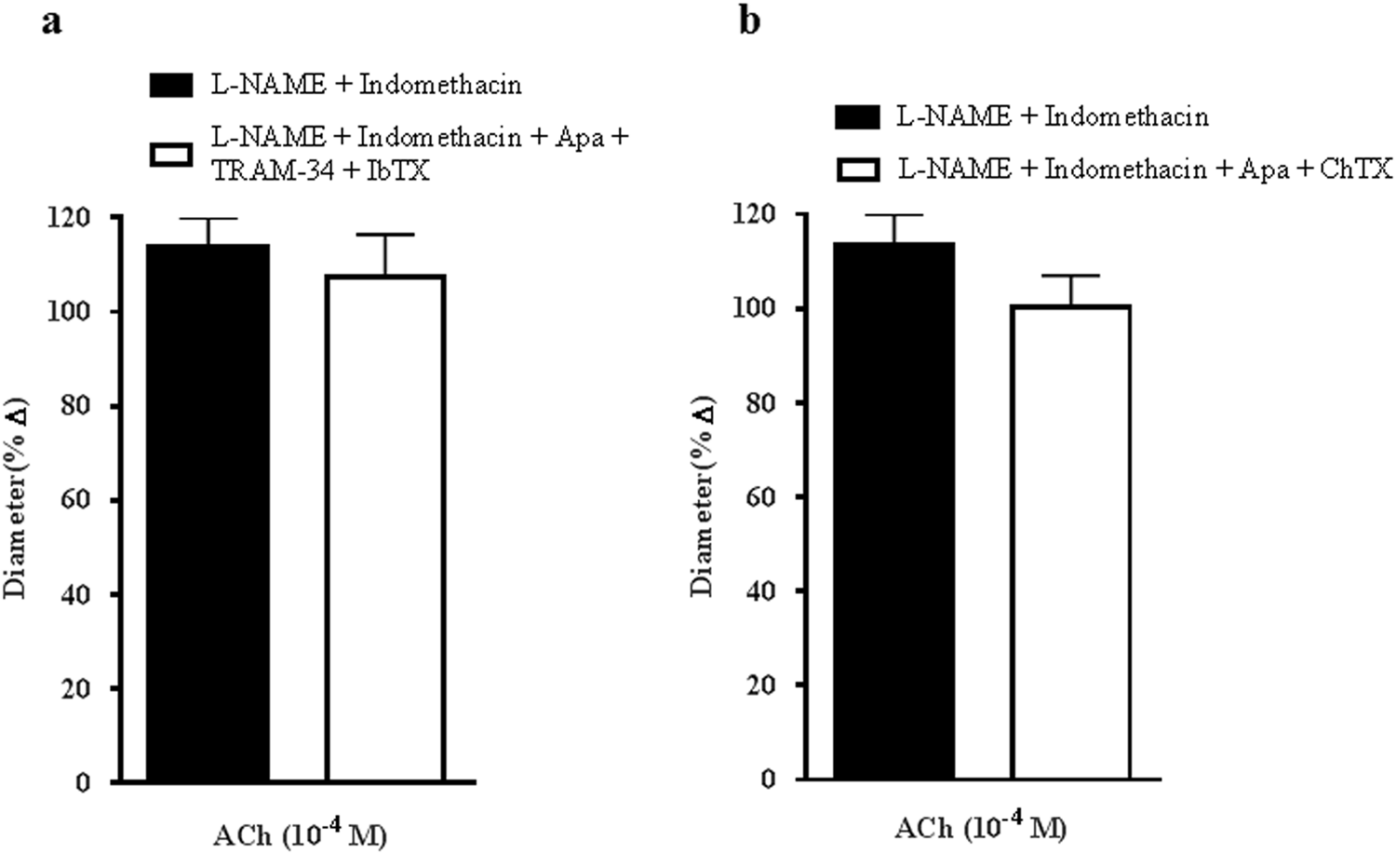
Calcium-activated potassium ion (K_Ca_) channels are not involved in mediating endothelium-dependent vasodilatory responses. (**a**) The combination blocking of the K_Ca_ channels with their respective specific blockers, Apa, TRAM-34 and IbTX and, (**b**) combined blockade with Apa and ChTX conferred negligible inhibitory effects on ACh-induced vasodilation. Values are expressed as mean ± s.e.m (n = 6 per group).

Several lines of evidence suggest that the calcium-activated potassium (K_Ca_) ion channels, comprising SK_Ca_, IK_Ca_ and BK_Ca_, are crucial components of the EDHF-mediated responses in many vascular beds^34, 35^. Therefore, to further determine the contribution of K_Ca_ channels in mediating EDHF-related vasodilation, all three channel subtypes were blocked with combination of highly specific inhibitors for each channel subtype consisting of Apa for SK_Ca_, TRAM-34 for IK_Ca_ and IbTX for BK_Ca_. This combination blocking demonstrated no inhibitory effect on ACh-induced vasodilation (L-NAME and indomethacin: 113.65 ± 6.35 ± % *vs* Apa + TRAM-34 + IbTX: 107.50 ± 8.90%), as depicted in Fig. 6a. To further validate this result, combination of Apa and ChTX were employed. Consistent with the former finding, this combination blockade also did not exhibit any significant inhibition (L-NAME and indomethacin: 113.65 ± 6.35% *vs* Apa and ChTX: 100.31 ± 6.70%) (Fig. 6b), confirming that the K_Ca_ channels are not involved in the ACh-induced vasodilation.

**Figure 7:**
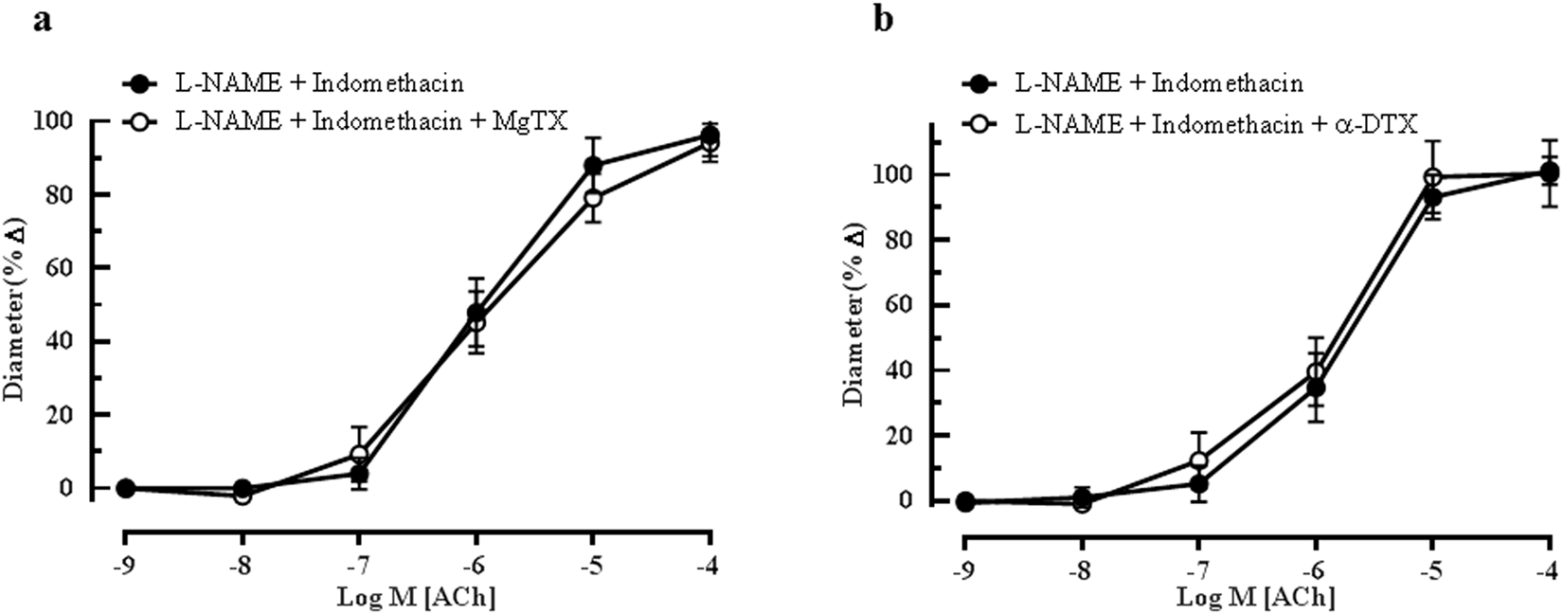
Effect of K_v_ channels inhibition on EDHF-mediated relaxation of ophthalmic artery. Blocking of vessels with (**a**) MgTX and (**b**) α -DTX demonstrated non-significant inhibitory effects on the ACh-mediated vasodilation. Values are expressed as mean ± s.e.m (n = 5–6 per group).

Apart from the K_Ca_ channels, there are several other potassium ion channels that are involved in EDHF-type responses in blood vessels. In our previous study, with the use of ChTX, margatoxin (MgTX) and alpha-dendrotoxin (α-DTX), we had demonstrated for the first time the involvement of the voltage-gated potassium ion channel K_v_1.6 in mediating endothelium-dependent vasodilation in the ophthalmic artery of wild type mice^24^. We followed up our initial findings and further sought to investigate if this channel subtype is also playing a role in mediating EDHF-type compensation in the lack of eNOS in this study. Unlike in the wild-type mice, the K_v_ channel had apparently no involvement in the cholinergic vasodilatory mechanisms in the ophthalmic artery of eNOS−/− mice, as evidenced by no inhibition of ACh-induced vasodilation in vessels incubated with MgTX (L-NAME and indomethacin: 96.29 ± 18.28% *vs* MgTX: 94.17 ± 17.1%) and α-DTX (L-NAME and indomethacin: 101.30 ± 19.14% *vs* α-DTX: 100.5 ± 19.33%) (Fig. 7a and b, respectively).

**Figure 8:**
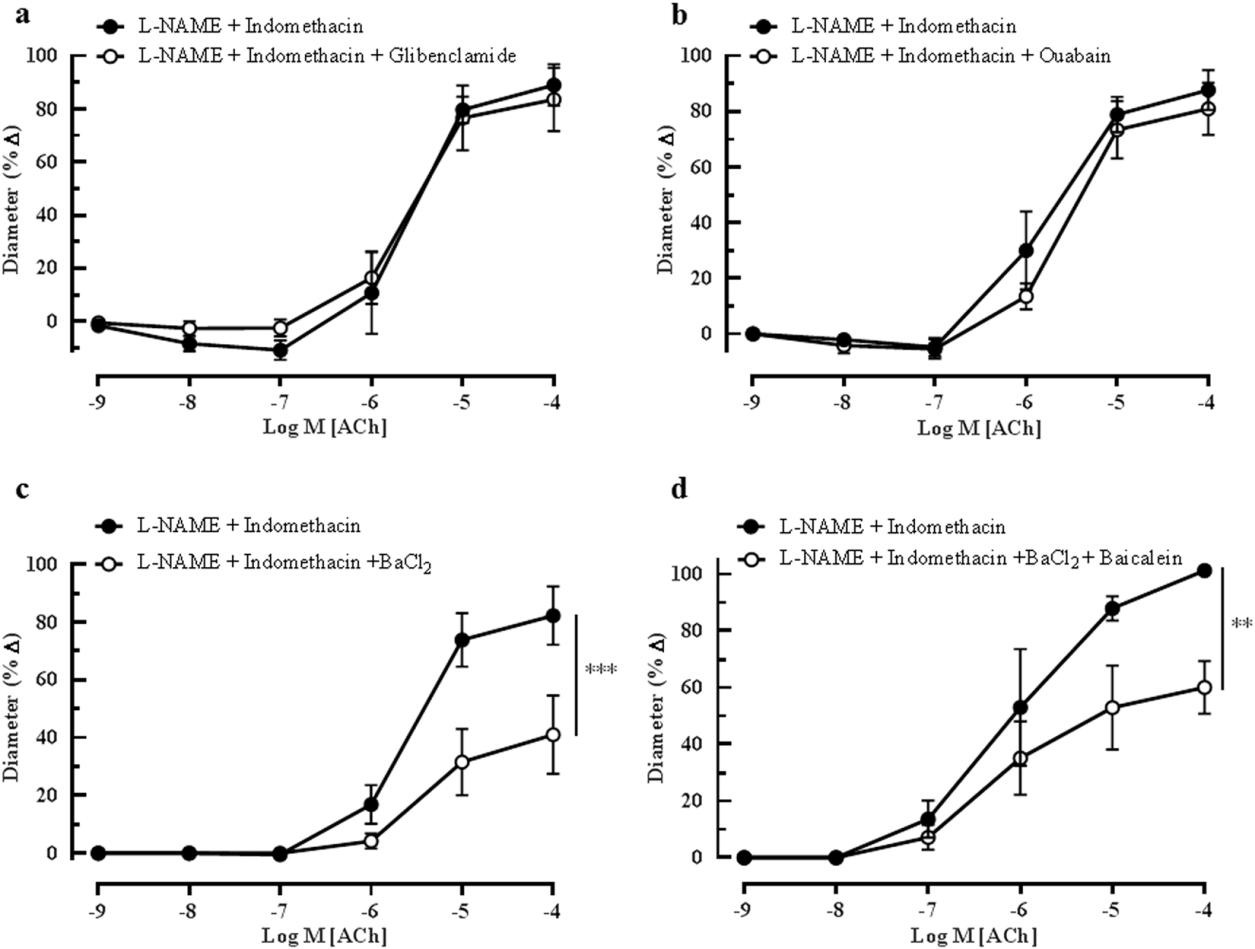
Cholinergic vasodilation of the mouse ophthalmic artery in the presence of potassium ion channels and Na^+^/K^+^-ATPase blockers. Incubation of the vessels with (**a**) glibenclamide and (**b**) ouabain elicited no significant inhibitory effects on ACh-mediated vasodilation. However, the inhibition of the (**c**) Kir channels with BaCl_2_ and, (**d**) combination blockade of Kir channels and LOX pathway with BaCl_2_ and baicalein caused a significant inhibition of dilation. Values are expressed as mean ± s.e.m (n = 4–6 per group; ***P < 0.001, L-NAME and indomethacin *vs* L-NAME and indomethacin and BaCl_2_, **P < 0.01, L-NAME and indomethacin *vs* L-NAME and indomethacin and BaCl_2_ and baicalein).

On the other hand, the blocking of K_ATP_ channel with glibenclamide and inhibition of the sodium-potassium pump (Na^+^/K^+^- ATPase) with ouabain demonstrated no significant inhibitory effects on endothelium-dependent vasodilation, which is in accordance with our previous results obtained in the wild type mice. Figure 8a and b show the ACh-mediated vasodilatory profiles of vessels incubated with glibenclamide (L-NAME and indomethacin: 88.99 ± 18.61% *vs* glibenclamide: 83.59 ± 16.61%) and ouabain (L-NAME and indomethacin: 87.68 ± 17.17% *vs* ouabain: 80.99 ± 16.33%), respectively. Conversely, preconstricted mouse opthalmic vessels relax to ACh *via* a mechanism that involves a barium-sensitive component because incubation with BaCl_2_ significantly blunted vasodilation (L-NAME and indomethacin: 82.19 ± 10.11% *vs* BaCl_2_: 40.97 ± 13.56%, P < 0.001), thus inferring contributions from the activation of Kir channels (Fig. 8c).

Taken together, these results demonstrate that only blockade of the LOX pathway and Kir channels attenuated ACh-induced vasodilation in the ophthalmic artery of eNOS−/− mice.

Therefore, to further determine whether both mechanisms are dependent on each other, as suggested previously^36, 37^, we tested responses to ACh before and after combined blockade with baicalein and BaCl_2_. Remarkably, this combination blockade significantly inhibited ACh-induced dilation (L-NAME and indomethacin: 101.25 ± 1.25% *vs* BaCl_2_ + baicalein: 60.03 ± 9.32%, P < 0.01), as depicted in Fig. 8d. However, this combination blockade did not reveal any additive blocking effect compared to respective single blockade of the LOX pathway with baicalein or Kir channels with BaCl_2_, suggesting dependency on each other.

## Discussion

The physiological integrity of an organism is maintained by complex networks of inter-connected cellular mechanisms. It comes as no surprise, therefore, that if these tightly regulated homeostatic pathways are breached due to disease conditions, dysfunction or even aging, nature has well-equipped the organism with several compensatory systems independent of the primary signal transduction pathways that strive to buffer the effects of deleterious changes^38–41^. Similarly in the ocular system, several lines of evidence have demonstrated the role of compensatory mechanisms in different parts of the eye that sustain near-normal functions in pathological conditions such as in diabetic retinopathy and anterior segment ischemia^42, 43^, as well as in aging^44^. In line of these investigations, the present study explored the identity of compensatory mechanisms that maintain ACh-mediated vasodilatory properties in the mouse ophthalmic artery when the predominant mediator, NO, is chronically deficient.

The primary observation of this study was that, despite the absence of nitric oxide-mediation, cholinergic vasodilatory responses are near-normal in the ophthalmic artery of eNOS-deficient mice due to an augmented role of EDHF, as demonstrated by the activation of the arachidonic acid-mediated LOX pathway and the Kir channels that maintained a moderating vasodilator influence. These findings differ remarkably from our previous findings in the ophthalmic artery of wild type mice where, in addition to the LOX metabolites and Kir channels, the major mechanisms that maintained ACh-mediated vasodilator responses comprised predominantly of the eNOS signalling pathway, CYP450 metabolites, gap junctions and the voltage-gated potassium channel, K_v_1.6^24^. It is also noteworthy that in this arterial bed of eNOS−/− mice, EDHF serves as the only back-up mechanism that subserves a key role in mediating endothelium-dependent dilation. Consistent with this finding, notable sole contribution of EDHF to ACh-mediated relaxation responses has been previously reported in the gracilis muscle arterioles and small mesenteric arteries of eNOS−/− mice^21, 45^. The enhancement and sole contribution of EDHF-mediated mechanisms may play crucial roles in maintaining near-normal responses in pathological conditions, such as diabetes, which may culminate in microvasculature complications namely retinopathy, neuropathy and nephropathy^46^. A similar phenomenon was also reported in the small mesenteric arteries of type 2 diabetic db/db mice, in which endothelium-dependent vasodilation was mediated entirely by EDHF^47^. Conversely, in some vascular beds such as the pial arterioles, coronary and small mesenteric arteries, eNOS deficiency was compensated by other NOS isoforms, namely nNOS^22, 48, 49^. On the other hand, a study by Chataigneau and co-workers demonstrated that several major arteries, such as the thoracic aorta and carotid arteries, were not able to compensate for eNOS deficiency in mice^19^. These discrepant results reflect the multifaceted heterogeneity in endothelial adaptive mechanisms to the loss of eNOS in vessels originating from different vascular beds.

Our previous studies employing wild type mice of different genetic backgrounds have demonstrated that cholinergic vasodilation of the ophthalmic artery is endothelium-dependent^24, 50^. Thus, in the current investigation, endothelium-denuded arterial preparations were also used to assess endothelium dependency of relaxations to ACh, the classic endothelium-dependent agonist in mice with congenital eNOS gene deficiency. The present findings in eNOS−/− mice correspond to our previous results in the wild type mice, where ACh-elicited relaxation was virtually abolished in ophthalmic arterial segments without intact endothelium and thereby, reiterating the obligatory role of the endothelium in the maintenance of vascular tone in this retrobulbar blood vessel. Consistent with the present result, several other studies also reported that endothelium removal abrogated agonist-induced relaxation in the pulmonary, saphenous, mesenteric and left anterior descending coronary arteries in eNOS mutant mice^51–54^. However, this may not be the case in other ocular blood vessels, such as in the bovine posterior ciliary arteries, where vasorelaxation remained unaffected after endothelial-denudation^55^.

The crucial finding in this study highlights the involvement of non-NO and non-prostanoid components that were able to maintain vasodilator responses to ACh *via* EDHF comprising the LOX metabolites of arachidonic acid, in particular *via* the 12-LOX pathway. Baicalein, which was used in the current study, is a known pharmacological inhibitor of 12/15-LOX^56, 57^ and importantly, it has been found that mice do not express 15- LOX and only express the leukocyte-derived 12-LOX^58, 59^. The complex array of mediators produced by the12-LOX pathway were found to be responsible for endothelium-dependent vasodilatory effect in arterial beds of different species, such as in the porcine and human coronary arteries^60, 61^, rabbit aorta^62^ and rat mesenteric and basilar arteries^63, 64^. It is important to highlight that the dilatory properties of blood vessels are different in healthy and disease conditions, where eNOS/NO bioactivity and functions are affected^65^. Therefore, it is not surprising that the 12/15-LOX signalling activity is elevated in vascular diseases associated with compromised bioavailability of NO^66^. Although 12-LOX-mediated mechanism contributed to L-NAME- and indomethacin-resistant relaxation in the ophthalmic artery of eNOS−/− mice, it remains to be determined which specific metabolite(s) is responsible for maintaining vasodilation. In mouse arteries, 12-hydroxyeicosatetraenoic acid (12-HETE), which is a product of endothelial cell 12-LOX metabolism, is identified as the predominant regioisomer^67^. Furthermore, 12-HETE functions as an EDHF, which induces smooth muscle hyperpolarization *via* the activation of large conductance K_Ca_ (BK_Ca_) channels in a variety of arteries such as in the rat basilar and mesenteric arteries^63, 64^, porcine coronary microvessels^61^, and human coronary arteries^68^.

In the present study, it is noteworthy that the LOX metabolites and Kir channels are dependent on each other because combination blocking of both components with their respective inhibitors demonstrated no additive effect on the inhibition of vasodilation. Similarly, in the rabbit mesenteric arteries, the combination blocking of the Kir and K_Ca_ channels with Ba^2+^ and ChTX, respectively, was only as effective as single blocking with either blocker, which indicated that these pharmacological agents act in series on the same pathway^69, 70^. It has been long recognized that arachidonic acid and its metabolites can interact with K^+^ channels to activate other modulatory signal transduction, although the exact mechanism(s) of action warrants further investigation^36, 37, 61^. A study by Volterra *et al*. has provided evidence that 12-LOX derivatives can directly activate a particular class of K^+^ channels to increase their open probability and for hyperpolarization to occur^71^. Furthermore, binding of certain lipid metabolites to Kir channels is known to induce allosteric conformational alterations in the channel pore, thereby driving it to an open state^72^.

The findings emerging from the current study differ remarkably from that of our previous study in the ophthalmic artery of wild type mice, where the LOX signalling mechanism seemed to be highly dependent on gap junctions. The discrepancy observed can be attributed to chronic impairment of eNOS and corresponding alteration in the EDHF-type relaxation, which is commonly seen in disease states. For example, mesenteric arteries of streptozotocin-induced diabetic rats exhibited different EDHF-related responses compared to normal controls due to defective gap junction signalling among other factors^73^. Additionally, decreased expression levels of connexins have been shown in vessels from mice with type 1 diabetes^74^.

**Figure 9:**
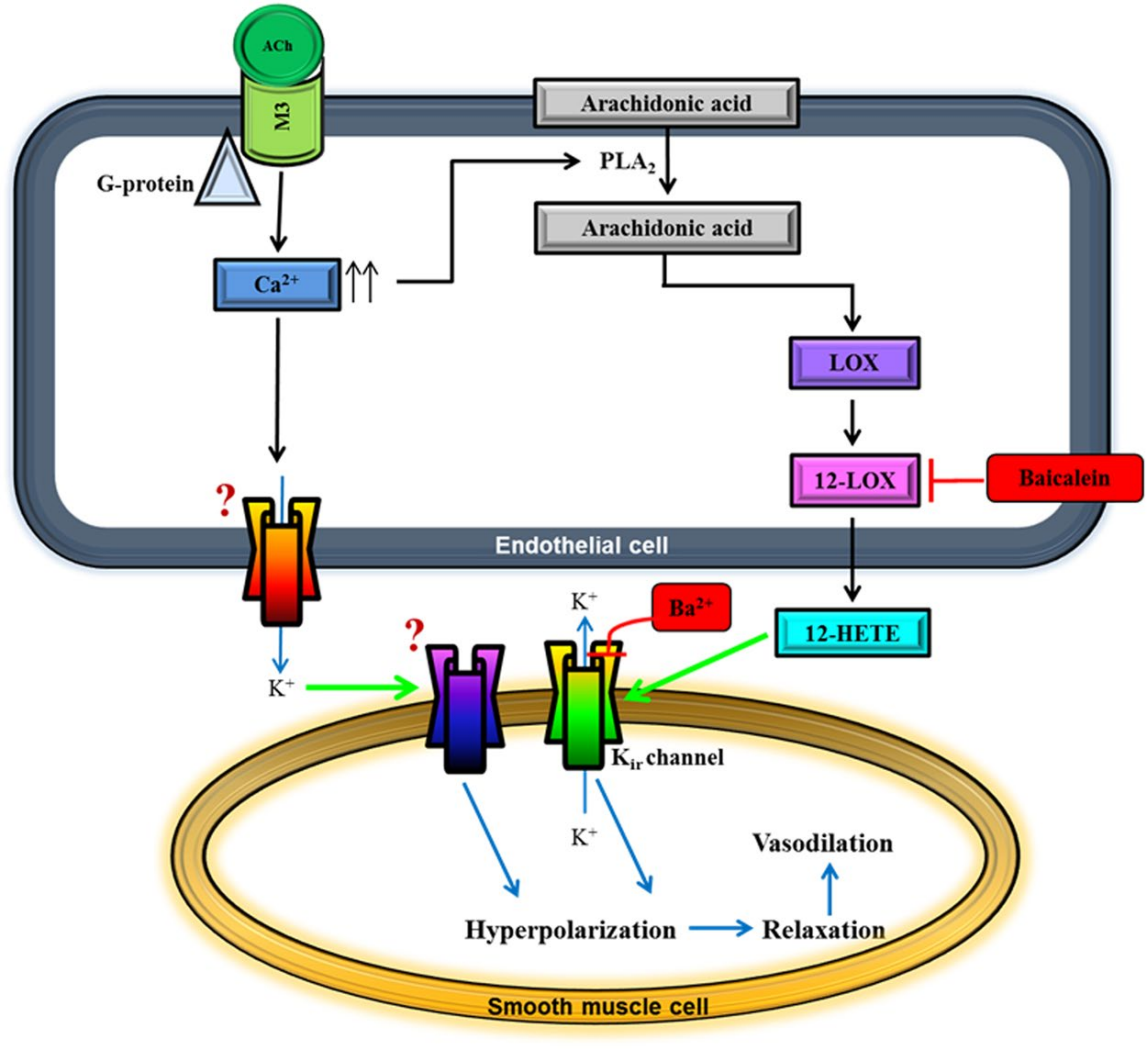
Proposed scheme for endothelium-dependent compensatory mechanisms responsible for mediating ACh-induced vasodilation in the ophthalmic artery of mice deficient in the eNOS gene. Endothelium-derived hyperpolarizing factor (EDHF)-mediated dilatory responses to ACh involve two mechanisms acting in parallel. In the first pathway, stimulation of the muscarinic ACh receptor (M_3_) coupled to G protein of class G_q_ on vascular endothelial cells mediates a transient increase in intracellular calcium, [Ca^2+^]i. Elevated endothelial [Ca^2+^]﻿i activates phospholipase A2 (PLA_2_) to release arachidonic acid (AA) from cell membrane. Free AA is metabolized *via* the 12-LOX pathway to produce relaxing factors, namely 12-HETE, which activates Kir channels on the smooth muscle. In the second pathway, it is hypothesized that elevated [Ca^2+^]﻿i also initiates activation of putative K^+^ channel(s) and release of K^+^ ions from the endothelial cell. These K^+^ ions diffuse to the adjacent vascular smooth muscle cell in concentrations sufficient to activate another putative K^+^ channel. Both pathways eventually cause hyperpolarization of the smooth muscle cell membrane and vasorelaxation. The precise identities of the putative channels hypothesized to be present in the ophthalmic artery of eNOS−/− mice that facilitate K^+^ efflux on the endothelial cell and are activated on the smooth muscle cell for downstream spread of hyperpolarization are unknown. Question marks represent unknown channels that remain to be identified. Inhibitor and blocker are indicated in red boxes. Green arrows indicate channel activation.12- HETE, hydroxyeicosatetraenoic acid; Kir, inward rectifying K^+^ channel.

Based on the findings of this study, a schematic representation of the hypothesized signalling pathways involved in the compensatory mechanisms that ameliorate the effects of impaired NO bioavailability is depicted in Fig. 9. In this proposed model, there are several important observations. First, it is hypothesized that there are at least two unidentified, novel channel subtypes with affinity for K^+^ on the endothelial and smooth muscle cell layers (indicated with question marks). This is because the conventional K^+^ channels usually implicated in EDHF-mediated responses, especially the K_Ca_ and K_v_ channels, were not responsible for efflux of K^+^ for hyperpolarization to occur, as evidenced by ineffective blocking with respective channel blockers, either alone or in combination. In line with this hypothesis comes a recent study by Coleman and co-workers suggesting the existence of a novel K^+^ channel with an unusual pharmacological fingerprint^75^. Therefore, the presence of a new channel that allows the passage of K^+^ is highly possible, although this hypothesis warrants further investigation. Second, the chemical identity of the putative LOX metabolites activating the Kir channels remains in question. In recent years, the state-of-the-art liquid chromatography (LC)/electrospray ionization (ESI)/tandem mass spectrometry (MS/MS) proteomic platform has emerged as a gold standard for detection of and unravelling specific identity of LOX-derived arachidonic acid metabolites^59, 67^. Albeit the usefulness and high accuracy of proteomic-based approaches, they are usually laborious and costly tasks, and are therefore, beyond the scope of the present study. Taken together, it is tempting to speculate that some previously unidentified and unexpressed compensatory mechanistic pathways may have become activated after selective eNOS gene deletion, which could explain the hypothesized putative K^+^ channels^76^.

In conclusion, this study provides evidence that the mouse ophthalmic artery possesses inherent compensatory mechanisms comprising EDHFs, which are able to retain normal cholinergic vasodilatory responses when the predominant relaxation mechanism *via* NOS/NO is chronically deficient. Most importantly, the findings in the ophthalmic artery disclose a previously undescribed compensatory cascade of events leading to vasodilation in response to ACh. The results of the present investigation assign a crucial regulatory role for LOX and its metabolites that act in concert with the Kir channels to orchestrate normal vasodilator responses. However, as above-mentioned, a number of significant gaps in our knowledge still remain to be investigated to provide for the missing links.

## Materials and Methods

### Animals

Male homozygous eNOS-deficient mice (C57BL/6J-NOS3tm1Unc, eNOS−/−) (The Jackson Laboratory, Bar Harbour, ME, USA), 3 to 7 months of age, were employed for the experiments. Mice were housed under specific pathogen-free in filter-topped cages and under standard conditions (temperature 23 ± 2 °C, relative humidity 55 ± 10% and 12 h light/ dark cycles), with access to regular rodent chow and water *ad libitum*. This study was approved by the German Federal Animal Rights Committee and conformed to the ‘The Association for Research in Vision and Ophthalmology’ (ARVO) statement for the use of animals in ophthalmic and vision research. Experiments were performed according to the guidelines of the German ‘Tierschutzgesetz’ and EU Directive 2010/63/EU for animal experiments.

### Drugs and Chemicals

The following drugs were used in this experiment: *N* ^ω^-nitro L-arginine methyl ester (L-NAME), indomethacin, acetylcholine hydrochloride (ACh), phenylephrine, catalase, baicalein, 18 alpha-glycyrrhetinic acid (18α-GA), ouabain, glibenclamide, barium chloride (BaCl_2_), (all purchased from Sigma-Aldrich Chemie GmbH, Steinheim, Germany), 17-octadecynoic acid (17-ODYA) and 1-[(2-chlorophenyl) diphenylmethyl]-1H-pyrazole (TRAM-34) (Tocris Bioscience, Bristol, UK), iberiotoxin (IbTX), charybdotoxin (ChTX) and apamin (AnaSpec Inc., Fremont, CA, USA), margatoxin (MgTX), α- and β-dendrotoxin (α- and β- DTX) (Alomone Labs, Jerusalem, Israel). Indomethacin, 17-ODYA, baicalein, glibenclamide and TRAM-34 were dissolved in dimethyl sulfoxide (DMSO). 18α-GA was dissolved in chloroform: ethanol (2:3) according to the manufacturer’s instructions. Apamin, ChTX, IbTX and L-NAME were dissolved in phosphate-buffered saline (PBS), whereas all other drugs were dissolved in distilled water.

### Vascular Preparation and Measurements of Vasoreactivity

The dissection and preparation of ophthalmic artery are as described previously^24^. Briefly, mice were sacrificed by carbon dioxide asphyxiation and, their eyes were rapidly removed together with retrobulbar tissues and placed in ice-cold Krebs-Henseleit buffer consisting of (in mM): 118.3 NaCl, 4.7 KCl, 2.5 CaCl_2_, 1.2 MgSO_4_, 1.2 KH_2_PO_4_, 25 NaHCO_3_, 11 glucose (Carl Roth GmbH, Karlsruhe, Germany). Next, the ophthalmic artery (luminal diameter between 80 and 150 μm) was isolated and dissected free of adherent connective tissues with fine-point tweezers and fine micro-scissors under a dissecting microscope, placed in an organ chamber filled with cold Krebs solution, cannulated and sutured onto micropipettes. Vessels were pressurized *via* the micropipettes to 50 mmHg under no-flow conditions and the chamber was continuously superfused with Krebs solution (37 °C, pH 7.4) and bubbled with 95% O_2_ and 5% CO_2_. The vessels were visualized using a video camera mounted on an inverted microscope and video sequences were captured to a personal computer for off-line analysis. Experiments were carried out after equilibration of the vessels for 30–40 minutes and vessels developed a moderate spontaneous myogenic tone during this period, as described in our previous studies^24, 77^. Viability of the arteries was assessed as a minimum of 50% constriction from the resting diameter in response to 100 mM KCl solution.

Subsequently, the arteries were preconstricted to 70–40% of the initial vessel diameter with the α_1_-adrenoceptor agonist phenylephrine. Phenylephrine was titrated to reach a similar preconstriction level before and after incubation with a blocker to standardize the average tone in each treatment group. The pre-treatment of the arteries with L-NAME may slightly constrict the arterial segments and in this circumstance, the phenylephrine concentration was adjusted to reach a similar preconstriction level in all experiments. In some arterial rings, the endothelium was gently removed by rubbing the intimal surface with a human hair, as described previously^50^. All reported drug concentrations represent final molar concentrations in the organ bath.

### Experimental Procedures

#### The first series of experiments investigated endothelium-dependency of the relaxation induced by ACh

Vasodilation was assessed in preconstricted endothelium-intact and endothelium-denuded ophthalmic arteries with ACh (10^−4^ M). Subsequently, the function of vascular smooth muscle was assessed with exogenous NO donor, sodium nitroprusside (SNP, 10^−4^ M).

#### The second set of experiments was conducted to determine the effect of inhibiting nitric oxide or prostanoids production on the vasoreactivity of ophthalmic artery

Cumulative concentration-response curves to ACh (10^−9^–10^−4^ M) were performed in pre-constricted vessels before and after incubation with L-NAME, (10^−4^ M), an arginine analogue that blocks NOS in a non-isoform-selective manner or indomethacin (10^−5^ M), a cyclooxygenase (COX) blocker. Each blocker was incubated for 30 min. The concentrations of L-NAME and indomethacin employed in the present study have been shown to effectively inhibit NOS and cyclooxygenase, respectively, in other vascular preparations, including the ophthalmic artery of wild type mice^24^.

#### To investigate the contribution of EDHF-like responses to ACh-mediated vasodilation, the third series of experiments were designed

Both L-NAME (10^−4^ M) and indomethacin (10^−5^ M) were present in the organ bath in addition to the inhibitors to examine the NO- and prostacyclin-independent vasodilatory responses, respectively. Vasodilatory responses of the ophthalmic artery to ACh (10^−9^–10^−4^ M) were tested before and after 30 minutes of incubation with the following pharmacological tools: catalase (1000 units/ml), a hydrogen peroxide (H_2_O_2_) inhibitor; 17-ODYA (10^−4^ M), a non-selective inhibitor of both ω-hydroxylation and epoxygenation of arachidonic acid *via* cytochrome P_450_ (CYP450); baicalein (10^−5^ M), a specific inhibitor of 12/15-lipoxygenase (12/15-LOX); 18α -GA (3 × 10^−5^ M), a gap junction blocker and ouabain (10^−4^ M), a sodium-potassium pump (Na+/K+ - ATPase) inhibitor.

#### The final series of experiments were carried out to determine the functional relevance and role of potassium ion channels in mediating vasodilatation responses in the ophthalmic artery

In the first protocol, vasodilatory responses of the vessels to ACh (10^−9^–10^−4^ M) were tested before and after 30 minutes of incubation with BaCl_2_ (10^−5^ M), an inwardly rectifying potassium (Kir) channel blocker and glibenclamide (10^−5^ M), a K_ATP_ channel inhibitor. In the second protocol, the role of calcium-activated potassium channels (K_Ca_) and voltage-gated potassium channels (K_v_) to ACh-induced vasodilation was investigated employing the following blockers; either alone or in combination: Apamin (10^−7^ M), a specific blocker of the small conductance K_Ca_ (SK_Ca_), TRAM-34 (10^−6^ M), a specific blocker of the intermediate conductance K_Ca_ (IK_Ca_), IbTX (10^−7^ M), a selective big conductance K_Ca_ (BK_Ca_) and ChTX (10^−7^ M), an inhibitor of both and big conductance K_Ca_ (BK_Ca_), which also blocks some of the K_v_ channels. In our previous study in the wild type mice, several K_v_ channel blockers were demonstrated to block the vasodilation to ACh, comprising MgTX, α- and β-DTX^24^. Thus, to check whether the same K_v_ channels are also responsible for mediating vasodilation in the eNOS−/− mice, arteries were incubated with these blockers for 30 min prior to concentration-response curve to ACh. In all K^+^ channel experiments, both NOS and COX inhibitors were present in the organ bath.

### Statistical Analysis

Data are presented as mean ± SEM, with *n* representing the number of mice per group, from which arteries were taken. Changes in vascular responses to the pharmacological agents tested are presented as percentage of diameter change from the initial pre-contraction levels. In single-dose experiments, comparisons were made by an unpaired (experiments of endothelial denudation) or paired (experiments with K_Ca_ blockers) two-tailed t-test. Comparisons were made using two-way ANOVA for repeated measures followed by the Bonferroni post-hoc test to detect individual differences. Differences were considered to be statistically significant when the *P* value was less than 0.05. Graph Pad Prism 6 software (GraphPad Inc., San Diego, USA) was used for statistical analyses.
